# Medical Students’ Creation of Original Poetry, Comics, and Masks to Explore Professional Identity Formation

**DOI:** 10.1007/s10912-021-09713-2

**Published:** 2021-11-15

**Authors:** Johanna Shapiro, Juliet McMullin, Gabriella Miotto, Tan Nguyen, Anju Hurria, Minh Anh Nguyen

**Affiliations:** 1 Department of Family Medicine, School of Medicine, UC Irvine Medical Center, University of California Irvine, rte 81, bldg. 200, rm 835, 101 City Dr. South, Orange, CA 92868 USA; 2 Department of Anthropology, University of California Riverside, Riverside, CA USA; 3 The Children’s Clinic, Long Beach, CA 90806 USA; 4 Department of Psychiatry and Human Behavior, School of Medicine, University of California Irvine, Orange, CA 92868 USA; 5 University of California Irvine, Irvine, CA USA

**Keywords:** Medical education, Arts in medical education, Professional identity formation, Poetry in medical education, Comics in medical education, Mask-making in medical education, Medical student wellness

## Abstract

**Introduction.** This study examines differences in students’ perceived value of three artmaking modalities (poetry, comics, masks) and whether the resulting creative projects offer similar or different insights into medical students’ professional identity formation. **Methods.** Mixed-methods design using a student survey, student narrative comments and qualitative analysis of students’ original work. **Results.** Poetry and comics stimulated insight, but masks were more enjoyable and stress-reducing. All three art modalities expressed tension between personal and professional identities. **Discussion.** Regardless of type of artmaking, students express concern about encroachments of training on personal identity but hoped that personal and professional selves could be integrated.

## Introduction

Over the past several decades, medical educators have worked diligently to integrate the arts and literature into medical school curricula (Shapiro et al. 2009; Lam et al. 2015; Haidet et al. 2016; Charon 2017; Kollmer-Horton 2019). This integration has utilized many different literary and visual/performing arts modalities; explored different curricular structures (e.g., elective vs. required); and experimented with different pedagogical approaches (exposure to existing works of art and literature vs. creation of original works). The goals of these efforts are multifaceted (Fitzgerald and Callard 2016; Wald et al. 2019), ranging from the instrumental (e.g., improve communication, advancing professionalism [Doukas, McCullough and Wear 2012; Blease 2016); the ethical (make more humane physicians [Nazario 2009]); the socially activist (help students appreciate issues of structural inequalities and the need for social justice in medicine [Gutierrez and DasGupta 2016]); the aesthetic (cultivate an appreciation for the human condition [Bleakley, Marshall and Bromer 2006]); and the empowering (helping students to find their voice [Thompson, Lamont-Robinson and Younie 2010]). Broadly speaking, sometimes arts and humanities have been conceived of as additive to medical training (remedying specific defects) and other times as integral (a value in and of themselves to future physicians [Bleakley 2015; Fitzgerald and Callard 2016]).

While the limited evaluation and assessment data available suggest that most of these endeavors are worthwhile according to criteria established by their proponents (Perry et al. 2011; Chen and Forbes 2014; Denhardt et al. 2016; Gowda et al. 2019), the plethora of content and methods make it difficult to differentiate aspects of value among the various approaches. Pursuing such issues, this study sought to determine a) whether there are differences in students’ perceived value of three artmaking modalities (poetry, comics, and masks); and b) whether the creative projects that result offer similar or different perspectives and insights into medical students’ professional identity formation and overall medical school experience.

### What artmaking reveals about professional identity formation

Artmaking can help us more fully understand core challenges that medical students face in building a professional identity. Kumagai (2012) notes three themes revealed through artmaking that are relevant to professional identity formation: identity dissonance, critiques of the hidden curriculum, and a loss of humanity. Identity dissonance, “the disconcerting internal experience of conflict between irreconcilable aspects of self” (Costello 2004), occurs when individuals struggle to meld the requirements of physician professional identity with already formed personal identities (Costello 2005; Creuss et al. 2015). It can produce significant emotional distress in medical students and lead to self-doubt and disillusionment.

Analysis of medical student poetry has shown that the medical school experience may create a perceived threat to self and personal values and a struggle to position oneself in humane, compassionate relationships with patients (Shapiro 2006). George and Green (2015) observe that, in their analysis of medical student comics, almost 50% portrayed their experiences by drawing on images of horror with the authors as victims of mistreatment. Work at the Uniformed Services University School of Medicine (Joseph et al. 2017; Stephens 2019; Stephens et al. 2019) linking mask-making to professional identity formation has consistently identified themes of role strain, isolation, burnout, and identity dissonance, although Shapiro et al.’s. (2018) research on preclinical medical student mask-making found more optimism that professional and personal identities could be reconciled.

### How artmaking contributes to professional identity formation

While analysis of the *content* of student artmaking reveals concerns about the personal cost of socialization into the culture of medicine, the *process* of creating art appears to exert a positive influence on professional identity formation. Whether the artmaking activity is writing poetry, creating a comic, or making a mask, the literature for each of these types of interventions cites enhanced skills in multiple areas essential for successful professional identity formation, including instrumental skills, humanistic orientation, self-care and personal growth, and social critiques of medical education, the healthcare system and beyond.

*Instrumental contributions* to professional identity resulting from medical student artmaking emphasize augmenting skills such as close observation, sustained attentiveness (Costello 2004; Green and Myers 2010; Pollak and Korol 2013; Cummings 2018) and communication (Cowen, Kaufman and Schoenherr 2016; Marthouret 2016; Shapiro et al. 2018; Mukunda et al. 2019). *Humanistic* outcomes of artmaking highlight aspects of patient care such as understanding patient experiences more deeply (Kumagai 2012; Jones et al. 2017; Cunningham et al. 2018); appreciation for multiple perspectives (Baruch 2017; Shapiro 2012; Charon, Hermann and Devlin 2016); and acceptance of uncertainty and ambiguity (Baruch 2017). Empathy and increased understanding of others’ suffering have also been consistently reported as a consequence of participation in creative activities (Shapiro and Stein 2005; Wald and Reis 2010; Chen and Forbes 2014; Garrie, Goel and Forsberg 2016; Wang et al. 2018).

Many studies have noted *self-care* (Schwartz et al. 2009; Small, Feldman and Oldfield 2017; Henderson et al. 2020) and *personal growth* (Jones et al. 2017) benefits for medical students who engage in artmaking practices. These include simple fun and relaxation but also refining their understanding of their own experiences (Kumagai 2012; Jones et al. 2017) and affirming personal values and expressing personal identity (Kumagai 2012). Others note increased capacity for reflection and self-awareness (Wear et al. 2012; Potash et al. 2014; Chen and Forbes 2014; Garrie et al. 2016; Cowen, Kaufman and Schoenherr 2016). Artmaking seems particularly useful in helping medical students cultivate the emotional awareness, processing, and regulation necessary in effectively practicing clinical medicine (Shapiro and Stein 2005; Kinsella and Bidinosti 2015; Green 2015; Cowen et al. 2016; Monk 2018). Artmaking fosters creativity and imagination as well (Potash et al. 2014)*.* Finally, artmaking provides medical students with a much-needed opportunity to offer *social critiques* of their education in medicine as well as the healthcare system in general (Broderick 2011; Kumagai 2012; Lake et al. 2015; Golub 2015; George and Green 2015; Courneya 2017; Cummings et al. 2018). In all these ways, artmaking builds essential skills for professional competency (Wald 2015; Wald and Weiss 2018).

Thus, analysis of medical student artmaking sheds light on the challenges posed by their efforts to become physicians while retaining core personal values and priorities (Cowen et al. 2016). Examining students’ evaluations of the artmaking experience itself provides insights more about the kinds of skill-building that occurs. Comparing the artmaking methods of poetry, masks, and comics, as we do in the present study, helps to nuance our knowledge of the process by which medical students become physicians, as well as the role the arts can play in supporting a resilient transition.

## Subjects and methods

### Subjects

Were ninety-four third year medical students out of a total class of ninety-five students. We did not collect demographic data by session, but the class was 52% female, with a mean age of twenty-eight years.

### Methods

This study occurred during an intersession, a weeklong break from clinical rotations about halfway through the clinical year, when students attend lectures and other supplemental training. All third-year students were required to participate in a two-hour session using one of three creative media (masks, comics, or poetry) to reflect on their experiences in this first year of clinical training. The study was reviewed and approved by the UCI Institutional Review Board HS#2017-3986.

Students could choose one of the three arts options or, if they did not indicate a preference, they were randomly assigned. For the mask-making, a total of thirty-four students participated, with thirty-one choosing this activity. For the comics, twenty-eight students participated and nine chose the activity. In the poetry group, thirty-two students participated, with eight students selecting this session. The prompt for all sessions read in part: *Today we would like to give you an opportunity to candidly express yourself. What would you like to tell us about yourself or about your experiences in medical school? Depending on your workshop, you will either create a comic, write a poem, or make a mask as a way of reflecting.*

Facilitators were three individuals with expertise in teaching medical students, physicians, and other health professionals how to create comics, masks, or poetry as ways of reflecting on professional experience. Each session commenced with a brief didactic presentation explaining the art form and its uses in medical education. For the remaining time, students created masks, comics, or poetry, then shared their work in dyads, and finally participated in a large group discussion about what it was like to create original art, and what it was like to share it. Students also wrote brief narrative comments reflecting on their project.

While students were required to participate in these sessions as part of their wellness curriculum and to complete an evaluation form, they were not required to participate in the research. The Study Information Sheet (SIS) explained that they consented to participate by turning in *either* their “work product” (mask, comics, poem), the reflective narrative, or both. Researchers stated in the SIS that the work products and narratives from the session would be treated confidentially and anonymously to respect the ethical issues involved in making such personal projects (Boydell et al. 2012; Parsons and Boydell 2012).

In the Mask group, we received six reflections without masks, and twenty-eight masks with narrative reflections. In the Comics group, we received nine reflections without comics, and eight comics with reflections. In the Poetry group, we received eleven reflections without poems, and twenty-six poems (some students wrote two poems) with reflections.

## Data analysis

### Quantitative data analysis

We conducted statistical analyses using SPSS v. 26 (IBM Corp, Armonk NY). We performed two ANOVA comparisons. One analysis compared all three modalities, and the second ANOVA examined the assigned/chosen data by comparing the following three groups: Verbal (assigned), Verbal (chosen), Masks (chosen) (it was impossible to include an assigned Mask group because so few students were in this category). The rationale for combining poetry and comics in the verbal groups was that both these modalities generally have a verbal, narrative concept, whereas masks focus on nonverbal, intuitive creation. Poetry and comics share an interest in cadence, rhythm and choice of word placement on a page. They both rely on a compactness and economy of words that conveys a host of meanings. Art, by contrast, is less analytical, more spontaneous, and can access tacit knowledge of which the creator is not consciously aware (Kumagai 2012).

### Qualitative data analysis

Researchers were formed into three teams, including perspectives from family medicine, psychiatry, anthropology, and psychology. Team members had prior experience analyzing either masks, poetry, or comics. To ensure consistency of approach, all team members underwent a two-hour training session to develop skills in analyzing all three modalities.

Each team was assigned an equal number of masks, poems, and comics. All researchers first independently reviewed their assigned work products, producing extensive written analyses of each item. They then reviewed the narratives provided by students commenting on their own work and integrated the student perspective into their interpretations. Next, they compared these interpretations with those of their teammate.

The entire research team met on three occasions for two hours each session to review the small group analyses, resolve or incorporate differences, and complete a summary grid of all findings, which was used as the basis for this paper. The team kept an extensive audit trail of all work.

We applied the concept of visual rhetoric to mask analysis and to the visual part of the comics analysis (Kress and van Leeuwen 1996). This method is designed to stimulate thinking about the specific messages conveyed through a piece of art. It includes detailed description of the artwork and examines dimensions of reality versus aspiration; saliency (noteworthy or significant aspects of the art); sectionality (how each section of the mask or panel is used); communication loci (eyes, mouth, ears in masks; how communication is expressed pictorially in comics); and emotions conveyed. For the comics analysis, we paid special attention to unique features such as the gutter, relationship between people and objects, and variations in written script.

We used a modification of the Listening Guide to analyze student narratives in all three conditions and written work products (i.e., content of poems and written language in comics) (Gilligan 2015; Mauthner 2017). This approach included listening for the story, consideration of multiple perspectives, and focusing on I-language to highlight identity issues. Grounded theory analysis (Corbin and Strauss 2008) informed our identification of themes.

### Quantitative results

Comparing the three modalities of poetry, comics, and masks, ANOVA analyses found that poetry and comics stimulated insight (self-reflection as a future doctor) for students (p=.05), while masks were more enjoyable (p =.03) and stress-reducing (p<.01) for students (Table 1). Comparing assigned vs. chosen status (Table 2), when verbal modalities were assigned, they were the least enjoyable for students, while masks (chosen) were most enjoyable (p=.02) and more relaxing and stress reducing (p<.01) than either chosen or assigned verbal modalities.

**Table 1:** Questionnaire scores by activity medium

	**Poetry**	**Comics**	**Masks**	F (df)	*p*
	(n=30)	(n=20)	(n=32)		
Q1: Was enjoyable	3.8 (1.1)	3.8 (1.0)	4.4 (0.8)	3.5 (2,79)	0.034*
Q2: Was a way to self-reflect & develop new insights about myself as a (future) doctor	3.9 (1.2)	3.5 (1.2)	3.1 (1.3)	3.1 (2,78)	**0.051†**
Q3: Was a way to self-reflect as a person & develop new insights about myself as a person	3.8 (1.2)	3.5 (1.1)	3.4 (1.3)	0.9 (2,78)	0.43
Q4: Was relaxing/stress-reducing	3.5 (1.2)	3.7 (1.0)	4.4 (0.9)	6.3 (2,78)	0.003**
Q5: Had stressful aspects	2.6 (1.4)	2.4 (1.6)	2.3 (1.2)	0.4 (2,77)	0.66
Q6: Is a way of promoting wellness & self-care	3.8 (1.0)	3.8 (0.9)	3.7 (0.9)	0.1 (2,77)	0.90

Table entries reported as mean (SD). Scores based on a 1-5 scale, with 5 being the highest. F statistics and p-values computed using one-way ANOVA. †p<0.1, *p<0.05, **p<0.01

**Table 2:** Questionnaire scores by Verbal/Nonverbal and Assigned/Chosen

	**Verbal (assigned)**	**Verbal (chosen)**	**Masks (chosen)**	F (df)	*p*
	(n=29)	(n=17)	(n=31)		
Q1: Was enjoyable	3.7 (1.1)	4.2 (0.7)	4.4 (0.8)	4.4 (2,75)	0.016*
Q2: Was a way to self-reflect & develop new insights about myself as a (future) doctor	3.7 (1.3)	3.9 (0.9)	3.1 (1.3)	2.9 (2,74)	0.064
Q3: Was a way to self-reflect as a person & develop new insights about myself as a person	3.7 (1.3)	3.8 (0.9)	3.4 (1.3)	0.6 (2,74)	0.53
Q4: Was relaxing/stress-reducing	3.6 (1.1)	3.7 (1.1)	4.4 (0.9)	6.3 (2,74)	0.003**
Q5: Had stressful aspects	2.6 (1.4)	2.2 (1.4)	2.3 (1.2)	0.6 (2,73)	0.56
Q6: Is a way of promoting wellness & self-care	3.7 (1.0)	4.1 (0.7)	3.7 (0.9)	1.6 (2,73)	0.22

Table entries reported as mean (SD). Scores based on a 1-5 scale, with 5 being the highest. F statistics and p-values computed using one-way ANOVA. *p<0.05, **p<0.01

### Qualitative findings

The primary questions asked in this paper focus on the perceived value of the three artmaking modalities and their role in professional identity formation. The following analysis and interpretations are a description of our findings that move between expressions within each of the modalities and identification of themes that allowed us to combine data across modalities.

#### Perceived value

All groups focused on experiences in medical school; however the Mask- making group often extended their reflection into more general areas of creativity. Both the Poetry and Comics groups stated that the most valuable part of the project was sharing their feelings and stories with others, hearing others’ stories in return, and discovering how much they shared in common. The students in the Poetry group commented on the value of being able to reflect on, think critically about and process their own and patients’ experiences and feelings and often mentioned achieving catharsis, clarity or insight after writing their poems. Similarly, students in the Comics group noted how the activity allowed them to express the importance of relationships with friends, family, classmates, mentors, and patients.

In the Mask group, although the instructions were identical to the other two groups, students sometimes chose medically-related themes and sometimes more general expressions of creativity. In their narratives, these students placed great emphasis on how mask-making helped them rediscover their creative side. Many noted the benefits of losing themselves in a focused task that had nothing to do with medicine. Whereas poetry and comics caused students to focus on difficult educational or clinical moments, in contrast many students perceived mask-making as a welcome distraction from immersion in the medical world. Several students referred to the exercise as a pleasant reversion to childhood. Many students suggested masks were just for “fun,” and denied that they were about anything in particular, but others explicitly noted the duality between presentational and real self.

## Main theme

**Fig. 1:**
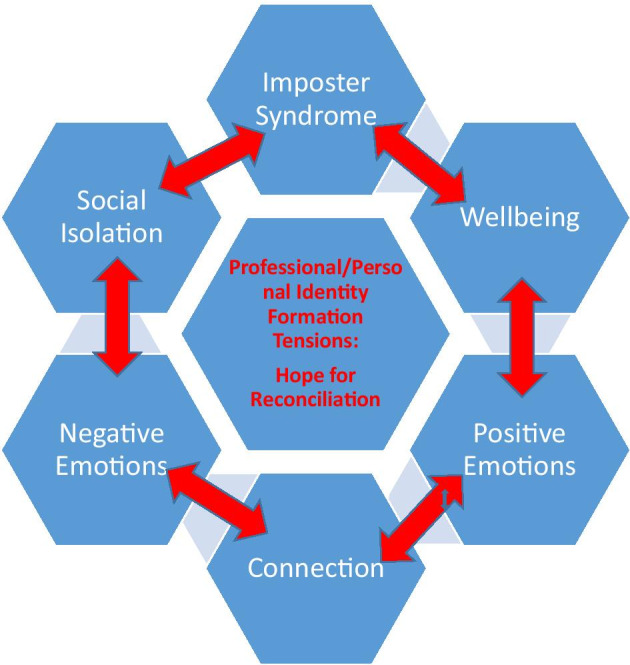
Conceptual Model - Tension between professional and personal identities: hope of reconciliation

In a systematic analysis of student comments and the artmaking projects themselves, the research team identified themes of professional identity formation that included imposter syndrome, yearning for personal wellness, emotional expression, and the need for human connection. We conceptualized these as representing a single overarching theme – **tension between professional and personal identities: hope of reconciliation** – to which all other sub-themes appeared related (Figure 1).

### Tension between professional identity formation and personal identity

Students in all groups saw the development of professional identity as difficult and demanding. Their artmaking activities revealed various levels of identity dissonance (Costello 2004), wherein who they were becoming as physicians was not as they imagined, thus leading to self-doubt, a perceived threat to personal values such as having compassionate relationships with patients, or concerns about burnout.

### Mask example^1^

Commenting on the challenges encountered in medical education, a student in the Mask group wrote, “There are two sides to every person and they are often intertwined. There is the self you present to others and your true self.” Masks sometimes appeared to express the concern that medical students were expected to be a perfect ideal, whereas they perceived themselves to be more complex and more stressed, but also richer. Another student (#88) from the Mask group wrote:

This mask represents two of the many states that I alternate between – in this case, that of spontaneity vs. premeditated. In a profession where the schedules and timelines are fixed and not flexible, I struggle to keep my spontaneity alive in the field of medicine.

In a moving disclosure, one Mask student (#72) commented,

The mask is a representation of how I feel at this point in my medical training. I feel pressure to speak positively of medicine and my experiences (yellow/sun colors) although I carry a lot of sadness in my head (blue/purple). My eye has been trained to see the best in situations (gold), but I have also been bruised by my experiences (black eye). I have felt anger (red cheek) and embarrassment (pink cheek) but must still speak sparkling words as I go through this journey.

Researchers observed that this mask was divided into thirds, with the expressive part (the mouth) bathed in sun colors, while the mental anguish remains hidden. They speculated that the rosy cheeks were an outward sign of glowing wellbeing, but disguised feelings of anger and shame.

### Poetry example^2^

Students who wrote poetry also struggled with what professionalism meant and what it might cost them. One student wrote in their poetry narrative, “[I’m] feeling a constant need to change who I am.” Another student-poet described their work as depicting “the struggles with conforming and becoming standardized.” Poem #43b (“Untitled”) presents professional identity as dehumanizing, threatening the personal values of the narrator. In this poem, the student witnesses the unexpected demise of a patient with a young family. The neurologist delivers the bad news briefly and dispassionately. The wife’s reaction is predictably anguished. The brief sympathy that the staff extends seems rote, designed more to protect them than to support the devastated wife. The team’s feelings are deeply buried behind literal and metaphorical masks. The student, determined to fulfill what they perceive as their professional – and human – obligation to share the wife’s pain, stays with her. In this case, the poet resists the professional role models, who appear to have betrayed their humanity and threatened values of paramount importance to the student. About this poem, the student wrote,

Recounting and narrative helped me process a trauma case. Med school changes us in ways good and bad…I hope that I can retain my empathy by the end of med school.

### Comics example^3^

Comic #18 represents how professional training negatively impacts the student’s initial sense of readiness and self-confidence. The excitement of being in medical school and having a readiness to learn are quickly overrun by the demands of medical school on the student’s time and body. Lectures turn into moments where the student dreams, not of their future as a physician, but of rest and food. The last panel shows the student at home and comforted by food. The student noted that their comic was intended to convey “the struggles” of being in medical school. Food and rest are secondary to class but in this comic show that without those basic needs they will struggle to become the physician that they imagined.

### Reconciliation of personal and professional identities

Poems, comics and especially masks often expressed the need to reconcile differing aspects of their personal and professional identities into a single authentic self that would not have to hide behind a mask. Projects from all three modalities advanced the aspiration that personal and professional identities could positively inform each other. Some projects emphasized that true professional identity should incorporate important personal values, service, good communication, and commitment to social justice.

### Mask example

Mask #78 depicts a calm mind (a peaceful blue) with a gem symbolizing the third eye of higher consciousness and enlightenment, crowned by a wreath of twigs and bark, suggesting a connection to the natural world. In the eye sockets are reflections of others who might be patients or simply people with whom the student wants to engage. Out of the mouth a rose emerges, perhaps representing love, surrounded by green, growing tendrils. The mask implies that professional identity should include human values of dedication to others, pursuit of personal growth, attainment of wisdom, acknowledgment of others’ suffering, careful listening and right speech.

### Poetry examples

Poems in general were more pessimistic that reconciliation was possible, but still often expressed the aspirational hope that all their traumas eventually will make sense. Poems often expressed the feeling that their personal joy and freedom is compromised, yet cling to the possibility that they can balance the demands of the educational process with personal values and commitment to patients and thus rekindle meaning. In Poem #44**,** for example, the writer catalogues the difficulties in their life, the sacrifices that they make, the ways they’ve changed, what they’ve endured. Nevertheless, in the final lines, this student concludes that being a doctor is still what they want, and that their relationships with patients confirm that “this is all I’ve ever wanted to be.”

### Comic example

Comic #21 depicts what the student experiences as a typical day, asking the question “What do you want to be when you grow up?” The second panel allows the reader to see the student walking to the hospital, stethoscope in one hand and a briefcase in the other, ready for their chosen profession. In a thought balloon, the student imagines the care that they will provide to a patient, talking with them, shaking the patient’s hand. As the student draws the events of the day, we see the phases of time passing through the window, sun rising, full sun, then stars. In each moment our student doctor is sitting in front of a computer, taking notes. During a patient visit, the student is portrayed looking at the patient file and taking notes on the computer. The student’s narrative describes their fears of what the profession might actually be like: “Working hard, being tired, and spending lots of time not interacting with people.” Yet, in the final panel, the student is still holding their stethoscope, implying their commitment to medicine remains intact.

## Supporting themes

The above examples demonstrate the broad challenges to professional identity formation. The following set of themes we describe provide deeper insight into questions of identity dissonance and the hidden curriculum. These themes range from imposter syndrome to emotions related to isolation and connection.

### Imposter syndrome

Because of their doubts about how professional identity connected with personal identity, students sometimes struggled with imposter syndrome (i.e., feelings of insecurity, uncertainty, self-doubt, unreadiness). The dualities inherent in many masks suggested that students felt they were “pretending” to be one way – competent, knowledgeable, compassionate, and cheerful – while in fact their feelings were entirely different. For example, we see in Mask #72 that the bright, shiny portrayal is very much at variance with the student’s actual feelings. “I Don’t Know How to Help” (Poem #35a) shows a student almost paralyzed with helplessness. Seeing all sides (patient-mother, daughter, resident, and student) of the problem, the author “knows what to do, but not how to help.” Daughter is overwhelmed, patient is frustrated, and resident feels he’s done what he can do. The student sees everyone’s helplessness, including their own, but there is no meaningful path forward they can identify that addresses any of the patient’s, daughter’s, or resident’s issues. The student ends up feeling like a failure. Returning to another reading of Comic #18, the student initially feels ready to participate in an anatomy dissection, but boring lectures, hunger, and sleep deprivation dampen their self-confidence; in the end the student passes the scalpel to a classmate.

### Emotions

Our analysis also suggested how students’ overall emotional state was related to professional identity formation. Students in the Poetry group explicitly focused on the opportunity to express their training-related emotions as a positive aspect of professional growth. As one student wrote, their goal was to “get more clarity for myself and others [about] the emotions involved in medical care.” Another student stated that they wanted to “express how tired/exhausted I am”; what resonated for them was the chance to “express my emotions.” A Comics student and a Poetry student each mentioned how important it was for them to “put the experience on paper” and “actually turn emotions into tangible words.” Another observed, “Poetry can be an expression of inner, indescribable feelings that can be therapeutic in itself and make us feel not alone.” Artmaking for these students provided clarity and connection with their emotions.

Common negative emotions expressed in all three conditions were exhaustion, sadness, and loneliness, reflecting students’ struggles with professional identity formation; although the presence of positive emotions found in all three activities, such as joy, connection with others, determination, and appreciation, suggested that students still found fulfillment in their lives. Overall, masks appeared to express more positive than negative emotions and were more optimistic than the poetry and comics projects. As one student wrote about her mask project, “While there are moments of self-restriction (i.e., self-doubt, frustration, disappointment etc.) there is still much beauty to life that manages to flourish and grow beyond the constraints placed.” One student in the Mask group expressed their confidence in these words: “Change is inevitable but always something to grow from.” Poetry and comics projects expressed more negative than positive emotions.

### Yearning for wellbeing/need for self-care

We often detected a longing in the creative projects for a better, happier life. Students were very aware of the sacrifices they were making to become physicians. In Mask #195, portraying a horse’s head, professional identity appears to stifle a free spirit wanting to race and roam. The student-creator wrote: “I often feel torn between my desire to become a doctor and the time commitments of medical school, and my passion for riding horses. Medical school has caused me to sacrifice a lot of my time/energy.” A haiku complained: “Why is it still dark/ When I wake up each morning?/ We all need more sleep!” By pluralizing the subject, the student makes an implicit critique of an exhausting, dehumanizing system of training. Comic #15 shows the student’s alarm going off at 4 am, the stars shining in her bedroom window. She responds to the alarm, “Kill me.”

Students in all three modalities expressed the need for self-care to regain peace, relaxation, and tranquility and to recapture a sense of hope, lightness, optimism, happiness and balance that existed prior to medical school. Masks in particular emphasized the importance of fun, playfulness, creativity and spontaneity. Mask #76 portrays a divided face, with what appear to be tears dripping from one eye and a mouth filled with pebbles. Yet the mouth is in the form of a surfboard, and the other side of the face depicts a tranquil and inviting ocean, suggesting that leisure activities could offer the student a healing respite. Students in the Poetry group mentioned keeping a perspective, spiritual beliefs, and mindfulness as ways of managing stress, overcoming imposter syndrome, and serving patients. Comics often included references to the importance of healthy eating, getting enough sleep, and managing stress. Comic #112 (not shown) is a story in which medical students gather together not only to study, but to eat well, and remind each other that they should not try to tackle the challenges of medical school alone.

The student narratives tended to support the quantitative results in terms of how they perceived the workshops as contributing to their personal wellness. Students experienced mask-making as a pleasant distraction or alternative to their clinical training, whereas poetry and comics were more of an opportunity to reflect on, examine, and delve deeper into often unsettling or disturbing clinical experiences. Mask-making students reported feeling “happy and relaxed,” described the experience as “cathartic,” an activity that allowed them “just to be,” and provided a “sense of release and satisfaction.” Many in the Mask group resisted the idea that their masks meant anything: "Painting is fun, don't ruin it by asking me to analyze it."

Writing poetry in particular received some negative comments (“relieved it’s over”; “put on the spot”; “sharing is uncomfortable”; “pressured – poetry isn’t for me”; “waste of time”), although these were balanced by more positive comments (“I felt creative,” “I felt much better,” “…I’d do it again any day”). One student captured their ambivalence by commenting “Relieved it’s over (proud to have written).” Students in the Poetry group frequently commented that the process of writing helped them organize their thoughts and attain deeper insight. As one described it, what most resonated was “committing words to paper and reflecting on the imagery of my experience to think critically about how other players thought and felt.” They went on to note the “catharsis of writing.” Another commented that “writing it out is very powerful,” while a third found that “recounting and narrative helped me process trauma case.”

Some of the Comics students commented that there was insufficient time to develop a narrative for the comic and also draw the story. Other students noted that they felt really stressed about drawing, but like the poetry they were glad that they were able to put their story on paper. As one student noted, “While creating the project, I felt reflective and challenged.” In observing the students after their comics drawing, many stood around in circles showing each other their comics. They laughed and commented on how good the others’ drawing or story was, then left with their comic. We are unsure if they were reluctant to share their work for the project or wanted to keep their comic to share with others.

### Isolation and connection

Isolation was a recurrent theme in both poems and comics that did not appear in masks. In a poignant haiku, despite three years with classmates, the student writes, “Nothing I’ve written/Could be shared with classmates/I barely know them.” In Poem #40, the student compares medical school to eating a stack of pancakes, from which one must be eaten every day, or the learner falls behind and can never catch up. The student catalogues all the family experiences they have missed because they have been faithfully “eating pancakes,” day in and day out. Ultimately, the student realizes they are sick of pancakes and especially sick of eating them alone. The poem becomes an appeal for others to join the student in helping to eat the pancakes, a yearning for a “pancake party” to break their isolation and restore a sense of enjoyment to their life.

Comic #15 “Living the Dream?” is a comment on the demands of medical education and what the student says are “the lows of third year.” The comic begins with a simple 11:12 pm text to their “mom” in which they make dinner plans for what she anticipates will be a short next day. The student’s next day begins at 4 am. She is scolded by a patient soon after for being on rounds so early. Eight hours later, in surgery she is scolded for not knowing the answer to a question about an artery that she is blocked from viewing. Another eight hours and she is told that she only needs to round on twenty-seven patients. Finally, the student arrives home at 10:37 pm, feeling like a “terrible daughter.” Her mother has take-out dinner ready and tries to encourage her daughter by reminding her that this is what she had always wanted to do and that as her mother, she is “so proud.” The student replies in a thought balloon, “Not exactly.”

Students perceived connection as both a need and a way of coping with some of the difficulties of medical school. We identified two types of connection – with nature and with people. A positive connection to the natural world (i.e., beaches, mountains, flowers, trees etc.), emerged in many of the mask projects, in contrast to no or almost no mention of nature in either comics or poetry. These masks tended to portray nature as a place of refuge from the world of medicine.

A majority of poems showed no connection with others. In these writings, there was no sense of partnership with other students or patients. The remaining poems showed either a slight or moderate connection with others (for example, in Poem #43b**,** the student connects with the patient’s wife and critiques the lack of connection that they see in other physicians). Masks were more evenly split, with a little over a third showing connection to other people. All the comics acknowledged connecting with others as a way of coping with the difficulties of medical school. They also revealed daily interactions and expectations that resulted in feelings of disconnection. In the Comics group, students wrote variously that “social interactions and friendships are vital in life,” “how important this person is to me” and touchingly noted that it was “quality not quantity of friendship” that mattered most.

## Discussion

The main results from our quantitative analyses indicated that mask-making was more fun, relaxing, stress-reducing, and a better source of self-care than either poetry writing or comics creating. Poetry writing seemed for some to be particularly frustrating, a finding that Marthouret (2016) also noted. On the other hand, students indicated that poetry and, to a lesser extent, comics, provided more insight into their professional lives, a finding also consistent with the literature (Wear et al. 2012). We suggest a relationship between these two findings, in that the less positive response to poetry may have resulted in part because students seemed to see poems as involving more personal disclosure, thus making them more vulnerable, especially in the sharing phase of the activity. Masks, from the students’ perspective, were simply a fun, relaxing activity that they perceived as revealing little or nothing about themselves and were therefore safer but less revelatory. In addition, poetry and comics served to immerse students more deeply in difficult clinical situations and stressful personal experiences, while masks appeared to students as a playful refuge from these things.

A perennial discussion in medical/health humanities focuses on whether this subject matter should be required or elective. Although not the purpose of this study, our results did shed light on this question. When we analyzed our data not by comparing art modalities but by chosen versus assigned across all modalities we found that, in comparison to assigned students, choosing students evaluated the activities to be significantly more enjoyable (p=0.008), more relaxing and stress reducing (p=0.03), and would be more likely to participate in similar activities in the future (p=0.04). Because addressing this question was not part of the initial design of this study, these results are confounded by the fact that almost all the students in the Mask condition chose to participate, whereas only nine of twenty-eight in the Poetry group, and eight of thirty-two in the Comics group elected these experiences. Nevertheless, these results confirm that when students have choice, they have a more enjoyable experience. They do not answer whether they benefit more educationally than those who are assigned, as there were no differences in their self-reported personal or professional insights resulting from participation in the workshop. Future research should pursue these and related questions.

Regarding the qualitative analysis, the overall theme we identified was the *tension between professional and personal identities.* We interpreted this theme as related to many sub-themes, specifically imposter syndrome, isolation, a longing for wellbeing and connection, and emotional expression. These subthemes contributed to or ameliorated this overarching tension. In different ways, they both explained students’ anxieties about becoming physicians and gave them hope.

All three modalities were concerned with the difficulty of professional identity formation in the third year of training, and the extent to which professional identity threatened personal identity (Green 2015; Joseph et al. 2017; Wald 2015). Overall, students in all three conditions found the development of a professional identity to be difficult, demanding, and full of personal sacrifice.

Many students seemed to feel that by pursuing the professional identity of a physician, they were in danger of losing their individuality and personal selves. They represented that their voices were often silenced, and they frequently felt like imposters, unable to fully conform to what they thought it meant to be a physician. Some felt coerced into a façade of cheerfulness, confidence, and certainty, whereas their subjective reality was more complex (Stephens et al. 2019). A few students saw professional and personal identities as informing each other (Rabow et al. 2013), aware that both needed to flourish in order to be an effective physician (Stephens 2019). Others wanted to ensure that professional identity also embodied their most precious personal values, such as service to others, compassion, and social justice.

An important component of professional identity formation that emerged in all three modalities was connection or its lack. Many student projects indicated a profound sense of disconnection from others, especially in the comics and poems. Masks showed more balance. This difference may be related to a split between conscious and unconscious, with students who used the analytic, narrative form of creating poetry or comics perceiving themselves to be isolated and alone, but with mask-makers having a more intuitive, tacit awareness of connection. Nevertheless, the overall conclusion was a disturbing sense of isolation and separateness.

As noted, masks showed a greater connection to nature than the other modalities, confirming an earlier study (Shapiro et al. 2018). This might have been partly because students could use natural materials such as feathers, bark, and shells to decorate their masks. However, other materials such as paint, ribbons, beads, and glass were not inherently evocative of nature. Also, the fact that the mask was in the shape of a face did not have anything to do with nature per se. It is more likely that in the intuitive space required for artistic creation, human closeness to the natural world sprang to mind more readily than when using verbal analysis for introspection.

A few other differences are worth noting. While most projects in all three modalities relied on the first person perspective, poetry experimented more with voice, which in turn may have produced greater empathy for different points of view, a finding reflected in existing literature (Chen and Forbes, 2014; Cowen et al. 2016; Shaffer et al. 2019). Comics focused more on relationships with family, friends, attendings, and patients, which may have allowed them to identify and think through interactions associated with the hidden curriculum. Overall, mask projects showed more positive emotions, as well as more connection to nature and to others. It is possible that something in mask-making itself, perhaps its more intuitive aspects, influenced the nature of the end product, so that the masks overall showed a more balanced, hopeful view of the medical student experience than either poetry or comics.

### Interpretations

One puzzle was the interpretive discrepancies between researchers and students in the mask-making condition. Some students tended not to see any deeper meaning in their work, while researchers often attributed profound insights to their projects. This was in striking contrast to comics and poetry, where interpretations of students and researchers were generally well-matched. We suspect that the differences in mask construal was because, in the absence of language, interpretation is necessarily more subjective. Also, in the absence of language, students’ unconscious issues may have surfaced without their explicit awareness.

### Limitations

Although we used a reflexive approach to heighten awareness of our own biases, assumptions, and subjectivities, our interpretations of these arts-based projects were inevitably influenced by our past training and life experiences. Further, since all teams interpreted all modalities, we may have unconsciously been more likely to find themes we’d identified in comics, for example, reemerging in poetry and/or masks. Finally, despite efforts, we were unable to coordinate with another institution, therefore our analyses were limited to the students of a single institution.

## Conclusions

This study is one of the first to compare the effects and purposes of different arts-related modalities used in medical education. We conclude that different modalities may affect students differently. In particular, less verbally based modalities, such as visual art, may be perceived as less personally revealing and therefore more relaxing and enjoyable. Modalities that involve some level of verbal reflection and analysis, such as comics, poetry or other forms of expressive writing, may be viewed as creating more vulnerability, and therefore more stressful, but also may offer more insights.

Overall, researchers found that similar themes emerged from the three arts modalities investigated. Students using all three modalities wrestled with the conflict between professional and personal identity formation; and struggled with isolation and a longing for more connection. However, an important difference was that poetry and comics tended to probe difficult clinical and educational situations, whereas masks overall conveyed more positive emotions, more perceived connectedness, and more general hopefulness.

All arts are not the same. They may have differing values to learners and different purposes in their implementation. More research is needed comparing different arts modalities both in terms of how students evaluated them and what we can learn about the students who engage with them.

## Figures and Tables

**Fig. 2:**
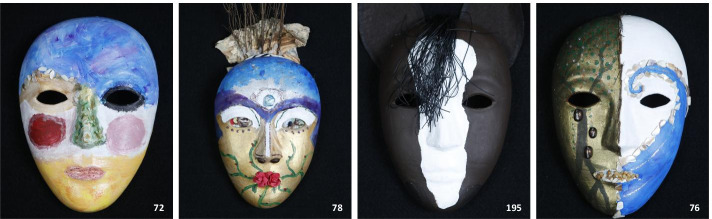
Examples of Student Masks

**Fig. 3:**
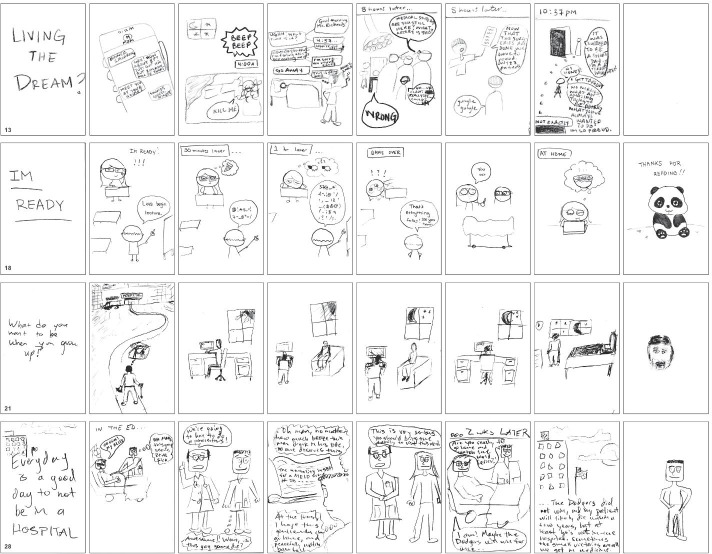
Examples of Student Comics

## Appendix examples of poems

### #43b Untitled

Young man, 3 kids, hits his head

Came into trauma, GCS 3

Team waited anxiously for CT scan

Abnormal black pooling of blood fills

the screen, as quickly as

dread pools in our stomach.

Intracranial hemorrhage, anoxic brain injury

Not going to survive. Nothing we can do

Neurologist broke the news

to wife looking terrified in the hallway

Grief, palpable, filled the hallway

Wails, chokes, sobs, gasps for breath

“Oh my god” repeated endlessly

Everyone disperses after 2 minutes of sympathy

in order to protect themselves

move on to the next trauma. There is no time.

Leaving behind the wife, still in shock,

and 2 staff to comfort her.

I stayed for longer, on purpose

until I felt tears in my eyes

listening to her say goodbye to her husband

She shouldn’t be alone.

I felt an obligation to share her pain.

I wanted to make sure I was still human

Could still feel emotions as we pass through

sterile hospital hallways and

greet fellow masks and suits of armor

Feelings of pain, weariness, anxiety buried deep within.

### #44 Untitled

Being a doctor is not for everyone

Training day and night

Leaving/coming home when there is no sunlight

Attempting to reinflate your patients’ souls with every interaction

When all you hope for is a smile, maybe a thank you to give you satisfaction

Looking to your family and friends to provide support

While you continue on the lonely road with emotions and desires all out of sorts

Yearning for a chance to feel whole and complete

But accepting that in a world of the sick and dying that that is an almost impossible feat

And so you cling to what makes you human: your love and empathy

Understanding that your patients are always coming, that you’re never free

Realizing that you’ve changed and trying to hit reset

Asking yourself where your body and soul have gone with every attempt

Being a doctor is not for everyone but I still think it is for me

Because despite and anguish and sadness I am my patients’ friend and keeper

And that is the only thing I’ve ever wanted to be

### #35A I don’t know how to help

“I have only one mother,”

Says the patient’s daughter

voice trembling

The list of problems she shows me on her phone

reveals this is not about the headache that brought them in

Each night she spends in the hospital

before leaving for work in the morning

“I don’t like to take medicine,”

the patient tells me

But each morning I find her

moaning in bed

She refused pain reliever the night before

She needs help to change, to go to the bathroom

“There’s nothing I can change,”

says the resident

We’ve run every test and have a few ideas

that our treatment should have helped by now

We can replete electrolytes

but otherwise we are not treating her at all

“How can you send her home like this?”

“I want to go home well, not be sent to some facility”

“She doesn’t need to be hospitalized”

“I don’t know how to help”

says the medical student

I think

I see the medical side – what to do for someone

without a clear problem, who refuses care

I see the daughter’s side – frustrated by a system

she thought she could trust

I see the patient’s side – not feeling herself,

clutching my hand

I’m stuck in the middle

I know what to do

But not how to help

### #40 Stack of pancakes

This is an ad for pancakes

I was told Med School was like a stack of pancakes

You need to each one each day so as to keep up

If you wait for it to pile up you’ll never finish

I ate the pancakes like I was supposed to

EVERY DAY. A pancake a day to keep the test takers away

And I guess they never told me that

I would get tired of pancakes

While my grandma was sick with the flu

I ate pancakes

When my cousin celebrated his 8^th^ birthday

I ate pancakes

When my mom called to ask why I didn’t come home

I ate pancakes

As I consumed pancakes, they consumed me

So this is an ad for pancakes

I’m looking for someone who wants to try pancakes

I’ve got loads to spare

I’ve learned that eating pancakes alone is just too great a task

If you have additional ingredients, a new idea

On how to make pancakes great again

Give it to me for my pancakes

Med School is like a tack of pancakes

Might as well have a pancake party

#### Endnotes

^1^All Mask examples cited are found in Figure 2

^2^All poetry examples cited are found in the Appendix

^3^All Comics cited are found in Figure 3

## References

[CR1] Baruch JM (2017). Doctors as Makers. Academic Medicine.

[CR2] Bleakley A (2015). *Medical Humanities and Medical Education : How the Medical Humanities Can Shape Better Doctors*.

[CR3] Bleakley A, Marshall R, Brömer R (2006). Toward an Aesthetic Medicine: Developing a Core Medical Humanities Undergraduate Curriculum. Journal of Medical Humanities.

[CR4] Blease C (2016). In Defence of Utility: The Medical Humanities and Medical Education. Medical Humanities.

[CR5] Boydell K, Tiziana SC, Katz A, Dow R, Brunger F, Parsons J (2012). Ethical Challenges in Arts-Based Health Research. International Journal of the Creative Arts in Interprofessional Practice.

[CR6] Broderick S (2011). Arts Practices in Unreasonable Doubt? Reflections on Understandings of Arts Practices in Healthcare Contexts. Arts & Health.

[CR7] Charon R (2017). *The Principles and Practice of Narrative Medicine*.

[CR8] Charon R, Hermann N, Devlin MJ (2016). Close Reading and Creative Writing in Clinical Education. Academic Medicine: Journal of the Association of American Medical Colleges.

[CR9] Chen, Isabel and Connor Forbes. 2014. “Reflective Writing and Its Impact on Empathy in Medical Education: Systematic Review.” *Journal of Educational Evaluation for Health Professions* 11 (20). 10.3352/jeehp.2014.11.20. 10.3352/jeehp.2014.11.20 PMC4309942 25112448

[CR10] Chute, Hillary. 2013. “Secret Labor: Sketching the Connection Between Poetry and Comics.” *Poetry Foundation* Jul/Aug. https://www.poetryfoundation.org/poetrymagazine/articles/70022/secret-labor.

[CR11] ----. 2016. *Disaster Drawn: visual Witness, Comics, and Documentary Form*. Massachusetts: Harvard University Press.

[CR12] Corbin, Juliet and Anselm Strauss. 2008. *Basics of Qualitative Research (3rd Ed.): Techniques and Procedures for Developing Grounded Theory*. California: SAGE Publications.

[CR13] Costello CY (2004). Changing Clothes: Gender Inequality and Professional Socialization. NWSA Journal.

[CR14] ----. 2005. *Professional Identity Crisis : Race, Class, Gender, and Success at Professional Schools*. Tennessee: Vanderbilt University Press.

[CR15] Courneya, Carol Ann. 2017. “Illustrating the Art of (Teaching) Medicine.” Edited by Susan Cox. *Cogent Arts & Humanities* 4 (1). 10.1080/23311983.2017.1335960.

[CR16] Cowen, Virginia S, Diane Kaufman, and Lisa Schoenherr. 2016. “A Review of Creative and Expressive Writing as a Pedagogical Tool in Medical Education.” *Medical Education* 50 (3): 311–19. 10.1111/medu.12878. 10.1111/medu.12878 26896016

[CR17] Cruess RL, Cruess SR, Donald Boudreau J, Snell L, Steinert Y (2015). A Schematic Representation of the Professional Identity Formation and Socialization of Medical Students and Residents: A Guide for Medical Educators. Academic Medicine.

[CR18] Cunningham Hetty, Delphine Taylor, Urmi A. Desai, Samuel C. Quiah, Benjamin Kaplan, Lorraine Fei, Marina Catallozzi, Boyd Richards, Dorene F. Balmer, and Rita Charon.2018. “Looking Back to Move Forward: First-Year Medical Students’ Meta-Reflections on their Narrative Portfolio Writings.” *Academic Medicine* 93 (6): 888–894. 10.1097/ACM.0000000000002102. 10.1097/ACM.0000000000002102 PMC5976514 29261540

[CR19] Cummings JR (2018). Comics and Medical Narrative: A Visual Semiotic Dissection of Graphic Medicine. Journal of Graphic Novels and Comics.

[CR20] Denhardt S, Apramian T, Lingard L, Torabi N, Arntfield S (2016). Rethinking Research in the Medical Humanities: A Scoping Review and Narrative Synthesis of Quantitative Outcome Studies. Medical Education.

[CR21] Doukas DJ, McCullough LB, Wear S (2012). Perspective. Academic Medicine.

[CR22] Fitzgerald D, Callard F, Whitehead A, Woods A, Atkinson S, Macnaughton J, Richards J (2016). *Entangling the Medical Humanities*.

[CR23] Garrie, Alaina J., Shruti Goel, and Martin M. Forsberg. 2016. “Medical Students’ Perceptions of Dementia after Participation in Poetry Workshop with People with Dementia.” *International Journal of Alzheimer’s Disease* 1–7. 10.1155/2016/2785105. 10.1155/2016/2785105 PMC4763010 26977333

[CR24] George DR, Green MJ (2015). Lessons Learned from Comics Produced by Medical Students. JAMA.

[CR25] Gilligan C (2015). The Listening Guide Method of Psychological Inquiry. Qualitative Psychology.

[CR26] Golub RM (2015). At What Cost?. JAMA.

[CR27] Gowda D, Curran T, Khedagi A, Mangold M, Jiwani F, Desai U, Charon R, Balmer D (2019). Implementing an Interprofessional Narrative Medicine Program in Academic Clinics: Feasibility and Program Evaluation. Perspectives on Medical Education.

[CR28] Green MJ (2015). Comics and Medicine. Academic Medicine.

[CR29] Green MJ, Myers KR (2010). Graphic Medicine: Use of Comics in Medical Education and Patient Care. BMJ.

[CR30] Gutierrez KJ, DasGupta S (2016). The Space That Difference Makes: On Marginality, Social Justice and the Future of the Health Humanities. Journal of Medical Humanities.

[CR31] Haidet P, Jarecke J, Adams NE, Stuckey HL, Green MJ, Shapiro D, Teal CR, Wolpaw DR (2016). A Guiding Framework to Maximise the Power of the Arts in Medical Education: A Systematic Review and Metasynthesis. Medical Education.

[CR32] Rebecca H, Hagen MG, Zaidi Z, Dunder V, Maska E, Nagoshi Y (2020). Self-Care Perspective Taking and Empathy in a Student-Faculty Book Club in the United States. Journal of Educational Evaluation for Health Professions.

[CR33] Jones EK, Kittendorf AL, Kumagai AK (2017). Creative Art and Medical Student Development: A Qualitative Study. Medical Education.

[CR34] Joseph K, Bader K, Wilson S, Walker M, Stephens M, Varpio L (2017). Unmasking Identity Dissonance: Exploring Medical Students’ Professional Identity Formation through Mask Making. Perspectives on Medical Education.

[CR35] Kinsella EA, Bidinosti S (2015). ‘I Now Have a Visual Image in My Mind and It Is Something I Will Never Forget’: An Analysis of an Arts-Informed Approach to Health Professions Ethics Education. Advances in Health Sciences Education.

[CR36] Kollmer Horton, Mary E. 2019. “The Orphan Child: Humanities in Modern Medical Education.” *Philosophy, Ethics, and Humanities in Medicine* 14 (1): 1. 10.1186/s13010-018-0067-y. 10.1186/s13010-018-0067-y PMC6322292 30616581

[CR37] Kress GR, Van Leeuwen T (1996). *Reading Images : The Grammar of Visual Design*.

[CR38] Kumagai AK (2012). Perspective: Acts of Interpretation: A Philosophical Approach to Using Creative Arts in Medical Education. Academic Medicine.

[CR39] Lake J, Jackson L, Hardman C (2015). A Fresh Perspective on Medical Education: The Lens of the Arts. Medical Education.

[CR40] Lam, Michael, Breanne Lechner, Ronald Chow, Leonard Chiu, Henry Lam, Marko Popovic, Milica Milakovic, Carlo DeAngeles, Edward Chow, and Blair Henry. 2015. “A Review of Medical Humanities Curriculum in Medical Schools.” *Journal of Pain Management* 289.

[CR41] Marthouret T (2016). Medical Humanities in the English Classroom: Building Students’ Professional Identity through Poetry. ASp - La Revue Du GERAS.

[CR42] Mauthner N (2017). *The Listening Guide Feminist Method of Narrative Analysis: Towards a Posthumanist Performative (Re) Configuration*.

[CR43] Monk J (2018). Go Home, Med Student: Comics as Visual Media for Students’ Traumatic Medical Education Experiences. AMA Journal of Ethics.

[CR44] Mukunda, Neha, Nazanin Moghbeli, Adam Rizzo, Suzannah Niepold, Barbara Bassett, and Horace M. DeLisser.2019. “Visual Art Instruction in Medical Education: A Narrative Review.” *Medical Education Online* 24 (1): 1558657. 10.1080/10872981.2018.1558657 PMC6394328 30810510

[CR45] Nazario RJ (2009). Medical Humanities as Tools for the Teaching of Patient-Centered Care. Journal of Hospital Medicine: An Official Publication of the Society of Hospital Medicine.

[CR46] Parsons JA, Boydell KM (2012). Arts-Based Research and Knowledge Translation: Some Key Concerns for Health-Care Professionals. Journal of Interprofessional Care.

[CR47] Perry M, Maffulli N, Willson S, Morrissey D (2011). The Effectiveness of Arts-Based Interventions in Medical Education: A Literature Review. Medical Education.

[CR48] Pollack, Alexia E., and Donna L. Korol. 2013. “The Use of Haiku to Convey Complex Concepts in Neuroscience.” *Journal of Undergraduate Neuroscience Education* 12 (1): A42–A48. https://www.ncbi.nlm.nih.gov/pmc/articles/PMC3852870/. PMC3852870 24319390

[CR49] Potash JS, Chen JY, Lam CLK, Chau VTW (2014). Artmaking in a Family Medicine Clerkship: How Does It Affect Medical Student Empathy?. BMC Medical Education.

[CR50] Rabow, Michael W., Carrie N. Evans, and Rachel N. Remen. 2013. “Professional Formation and Deformation: Repression of Personal Values and Qualities in Medical Education.” *Family Medicine* 45 (1): 14–18. https://pubmed.ncbi.nlm.nih.gov/23334962/. 23334962

[CR51] Schwartz AW, Abramson JS, Israel WI, Accordino R, Ronan EJ, Rifkin MR (2009). Evaluating the Impact of the Humanities in Medical Education. Mount Sinai Journal of Medicine: A Journal of Translational and Personalized Medicine.

[CR52] Shaffer, Victoria A., Jennifer Bohanek, Elizabeth S. Focella, Haley Horstman, and Lise Saffran. 2019. “Encouraging Perspective Taking: Using Narrative Writing to Induce Empathy for Others Engaging in Negative Health Behaviors.” Edited by Paula Pérez-Sobrino. *PLOS ONE* 14 (10). 10.1371/journal.pone.0224046. 10.1371/journal.pone.0224046 PMC6793876 31613906

[CR53] Shapiro J (2006). Listening to the Voices of Medical Students in Poetry: Self, Patients, Role-Models and Beyond. Journal of Poetry Therapy.

[CR54] ----. 2012. “Whither (Whether) Medical Humanities? The Future of Humanities and Arts in Medical Education.” *Journal for Learning through the Arts: A Research Journal on Arts Integration in Schools and Communities* 8 (1). 10.21977/d98111796.

[CR55] Shapiro, Johanna, and Howard Stein. 2005. “Poetic License: Writing Poetry as a Way for Medical Students to Examine Their Professional Relational Systems.” *Families, Systems, & Health* 23 (3): 278.

[CR56] Shapiro J, Coulehan J, Wear D, Montello M (2009). Medical Humanities and Their Discontents: Definitions, Critiques, and Implications. Academic Medicine.

[CR57] Shapiro J, Youm J, Heare M, Hurria A, Miotto G, Nguyen B-N, Tan N, Simonson K, Turakhia A (2018). Medical Students’ Efforts to Integrate and/or Reclaim Authentic Identity: Insights from a Mask-Making Exercise. Journal of Medical Humanities.

[CR58] Small LC, Feldman LS, Oldfield BJ (2017). Using Narrative Medicine to Build Community across the Health Professions and Foster Self-Care. Journal of Radiology Nursing.

[CR59] Stephens MB (2019). Behind the Mask: Identity Formation and Team Building. The Annals of Family Medicine.

[CR60] Stephens MB, Bader KS, Myers KR, Walker MS, Varpio L (2019). Examining Professional Identity Formation through the Ancient Art of Mask-Making. Journal of General Internal Medicine.

[CR61] Thompson, Trevor, Catherine Lamont-Robinson, and Louise Younie. 2010. “‘Compulsory Creativity’: Rationales, Recipes, and Results in the Placement of Mandatory Creative Endeavour in a Medical Undergraduate Curriculum.” *Medical Education Online* 15 (1). 10.3402/meo.v15i0.5394. 10.3402/meo.v15i0.5394 PMC3037267 21321668

[CR62] Wald HS (2015). Professional Identity (Trans)Formation in Medical Education. Academic Medicine.

[CR63] Wald HS, McFarland J, Markovina I (2019). Medical Humanities in Medical Education and Practice. Medical Teacher.

[CR64] Wald HS, Reis SP (2010). Beyond the Margins: Reflective Writing and Development of Reflective Capacity in Medical Education. Journal of General Internal Medicine.

[CR65] Wald, Hedy S., and Barrett Weiss. 2018. “Making it ‘More Real’: Using Personal Narrative in Faculty Feedback to a Medical Student’s Reflective Writing–An Illustrative Exemplar.” *MedEdPublish* 7 (3): 33. 10.15694/mep.2018.0000171.1 10.15694/mep.2018.0000171.1 PMC10701800 38074617

[CR66] Wang R, Houlden RL, Catherine HY (2018). Graphic Stories as Cultivators of Empathy in Medical Clerkship Education. Medical Science Educator.

[CR67] Wear D, Zarconi J, Garden R, Jones T (2012). Reflection in/and Writing. Academic Medicine.

